# Bile acids and nonalcoholic fatty liver disease: Molecular insights and therapeutic perspectives

**DOI:** 10.1002/hep.28709

**Published:** 2016-08-04

**Authors:** Juan P. Arab, Saul J. Karpen, Paul A. Dawson, Marco Arrese, Michael Trauner

**Affiliations:** ^1^ Department of Gastroenterology, School of MedicinePontificia Universidad Católica de ChileSantiagoChile; ^2^ Division of Pediatric Gastroenterology, Hepatology and Nutrition, Department of PediatricsEmory University School of MedicineAtlantaGAUSA; ^3^ Division of Gastroenterology and Hepatology, Department of Internal Medicine IIIMedical University of ViennaViennaAustria

## Abstract

Nonalcoholic fatty liver disease (NAFLD) is a burgeoning health problem worldwide and an important risk factor for both hepatic and cardiometabolic mortality. The rapidly increasing prevalence of this disease and of its aggressive form nonalcoholic steatohepatitis (NASH) will require novel therapeutic approaches to prevent disease progression to advanced fibrosis or cirrhosis and cancer. In recent years, bile acids have emerged as relevant signaling molecules that act at both hepatic and extrahepatic tissues to regulate lipid and carbohydrate metabolic pathways as well as energy homeostasis. Activation or modulation of bile acid receptors, such as the farnesoid X receptor and TGR5, and transporters, such as the ileal apical sodium‐dependent bile acid transporter, appear to affect both insulin sensitivity and NAFLD/NASH pathogenesis at multiple levels, and these approaches hold promise as novel therapies. In the present review, we summarize current available data on the relationships of bile acids to NAFLD and the potential for therapeutically targeting bile‐acid‐related pathways to address this growing world‐wide disease. (Hepatology 2017;65:350‐362)

**Abbreviations**
ABC: ATP‐binding cassette
Apo: apolipoproteins
ASBT: ileal apical sodium‐dependent bile acid transporter
BA: bile acid
BAT: brown adipose tissue
Bsep: bile salt export pump
CA: cholic acid
CDCA: chenodeoxycholic acid
CYP7A1: cholesterol 7α‐hydroxylase
DCA: deoxycholic acid
FA: fatty acid
FGF: fibroblast growth factor
FGFR4: FGF receptor 4
FXR: farnesoid X receptor
FXR‐KO: FXR‐knockout
GLP‐1: glucagon‐like peptide 1
GM: gut microbiota
HCC: hepatocellular carcinoma
HDL: high‐density lipoprotein
HFD: high‐fat diet
HOMA: homeostasis model assessment
LCA: lithocholic acid
LDL: low‐density lipoprotein
LDLR: LDL receptor
LPL: lipoprotein lipase
NAFLD: nonalcoholic fatty liver disease
NAS: NAFLD Activity Score
NASH: nonalcoholic steatohepatitis
OCA: obeticholic acid
OSTα‐OSTβ: heteromeric organic solute transporter alpha‐beta
PPARα: peroxisome proliferator‐activated receptor alpha
SCD1: stearoyl CoA desaturase 1
SHP: small heterodimer partner
SLC: solute carrier
SR‐BI: scavenger receptor class B type I
SREBP‐1c: sterol‐regulatory element‐binding protein‐1c
T2DM: type 2 diabetes mellitus
TGR5: G‐protein‐coupled bile acid receptor 1 (GPBAR1)
UDCA: ursodeoxycholic acid
VLDL: very‐low‐density lipoprotein

Nonalcoholic fatty liver disease (NAFLD) is an emerging health problem worldwide, affecting between 25% and 30% of the general population.^1^ NAFLD refers to a spectrum ranging from noninflammatory isolated steatosis to nonalcoholic steatohepatitis (NASH), which is characterized by steatosis, necroinflammatory changes, and varying degrees of liver fibrosis.^2^ Patients with NAFLD exhibit an increased risk of death linked to type 2 diabetes mellitus (T2DM) and cardiovascular risk factors,^3^ and those with NASH have also an increased liver‐related mortality attributed to the progression to cirrhosis and hepatocellular carcinoma (HCC).^1^ Recent studies have highlighted the prognostic relevance of the presence of liver fibrosis in determining long‐term liver‐outcomes of NAFLD.^4^ As NAFLD became increasingly common in the developed world over the last decade, NASH has risen as a cause of chronic liver disease and is currently the second‐leading etiology of cirrhosis among adults awaiting liver transplantation in the United States.^5^ Moreover, emerging data suggest that the recent increase in the incidence of HCC is driven by NAFLD, particularly in Western countries.^6^

Currently, promotion of lifestyle changes in diet and exercise habits as well as control of comorbidities (i.e., T2DM and dyslipidemia) remain the cornerstone of NAFLD management.^7^ The drug armamentarium to treat NAFLD/NASH is today rather limited,^8^ although new approaches are being intensively explored.^9^ In this context, bile acid (BA) derivatives and compounds that influence BA‐related signaling pathways are emerging as potentially useful therapeutic agents for NAFLD and NASH.^10^, ^11^, ^12^ In the present review, we provide a summary of current knowledge on the role of BAs in NAFLD/NASH and present new insights into the possible approach of targeting BA‐related pathways in the treatment of this serious global health problem.

## Bile Acids as Signaling Molecules

BAs are amphipathic steroid molecules synthesized in the liver from cholesterol and excreted into bile as one of its main components. BAs (amino‐acyl‐conjugates of the primary BAs, cholic acid [CA] and chenodeoxycholic acid [CDCA], and their secondary metabolites) are actively secreted by the hepatocyte into the canaliculus serving as the main driving force for bile production.^13^ BAs, along with other biliary constituents, empty into the small intestine, where they function in the emulsification and absorption of dietary fat, cholesterol, and fat‐soluble vitamins. After reaching the terminal ileum, BAs are almost completely (∼95%) absorbed by an active uptake mechanism. This limits loss in the feces to approximately 0.2‐0.6 g/day, which is balanced by the daily synthesis of BAs. In the distal small intestine and colon, the primary BAs, CA and CDCA, undergo deconjugation and dehydroxylation by resident bacteria, resulting in the formation of secondary BAs (i.e., deoxycholic acid [DCA] and lithocholic acid [LCA]). These secondary BAs can be reabsorbed passively and constitute a portion of the total BA pool that cycles in the enterohepatic circulation. As a result of their efficient hepatic extraction, the concentration of BAs in the systemic circulation and peripheral tissues is extremely low, with only small incremental rises in postprandial periods (for a recent review of BA metabolism, please see Dawson and Karpen^14^).

**Figure 1:**
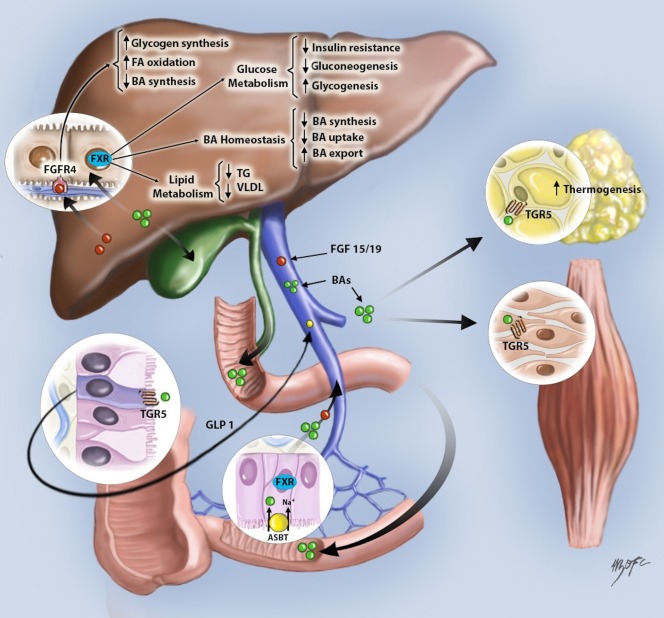
Schematic representation of the action of BAs as signaling molecules in several tissues: The nuclear receptor, FXR, is activated by BAs in the liver and has several downstream effects, including inhibition of lipogenesis, decreased BA synthesis, decreased gluconeogenesis, and increased insulin sensitivity. BAs also activate the Takeda G‐protein‐coupled receptor 5 (TGR5) in muscle and adipose tissues, increasing thermogenesis and energy expenditure. Also, activation of TGR5 in the intestine promotes GLP‐1 release from L cells, which, in turn, promotes insulin release from pancreatic β‐cells. In the terminal ileum, and after BA uptake by ASBT, FXR also stimulates production of FGF15 (mice) or 19 (human) that, upon binding to FGFR4 in liver cells, represses BA synthesis and promotes hepatic glycogen storage and FA oxidation. Abbreviation: TG, triglycerides.

For many years, it was thought that the functions of BAs were largely limited to stimulating hepatic bile flow and biliary excretion, and aiding digestion and absorption of fats from the intestinal lumen. However, studies over the past two decades (recently reviewed by Chiang^15^) led to the understanding that BAs may function as signaling molecules through a variety of receptors to regulate their own synthesis as well as other metabolic processes, such as glucose, lipid, and energy homeostasis.^16^ The regulatory actions of BAs are mediated through specific BA‐activated receptors, including members of the nuclear receptor superfamily (farnesoid X receptor [FXR; NR1H4], vitamin D receptor [NR1I1], and pregnane X receptor [NR1I2]), and members of the G‐protein‐coupled receptor superfamily (TGR5 and sphingosine‐1‐phosphate receptor 2).^11^ These receptors are expressed by tissues within the enterohepatic circulation, but also beyond the liver and gastrointestinal system, where they mediate systemic actions of BAs.^16^ Much of our current understanding of the regulatory actions of BAs comes from studies dealing with FXR and TGR5, although the metabolic actions of BAs likely involved other pathways as well (Fig. 1). These BA‐regulated receptors may influence NAFLD development and progression at multiple levels and are summarized below.

## Bile Acids and FXR: Regulation of Glucose and Lipid Metabolism

FXR, originally named for its ability to bind farnesoid, remained as an “orphan” receptor until 1999, when three groups identified BAs as the natural ligands for FXR.^17^, ^18^, ^19^ Shortly thereafter, studies using FXR‐knockout (FXR‐KO) mice demonstrated a major role of this receptor in controlling BA homeostasis and suggested that FXR regulatory functions extended beyond BAs to both glucose and lipid metabolism.^11^, ^16^, ^20^

With regard to the involvement of FXR in glucose metabolism (see previous works^21^, ^22^ for details), studies using whole‐body FXR‐KO mice showed that these mice exhibit decreased insulin sensitivity. Conversely, treatment with the selective, nonsteroidal FXR agonist, GW4064, improved insulin resistance and glucose homeostasis in obese *ob/ob* and diabetic *db/db* mice. Interestingly, plasma glucose levels were reduced in FXR‐KO mice after administration of adenovirus expressing a constitutively active FXR, but not when FXR‐KO mice were treated with GW4064, indicating that modulation of insulin action is dependent upon the presence of the receptor.^23^ In line with a role for BA in insulin resistance, serum levels of CDCA, CA, and DCA in humans are negatively associated with insulin sensitivity.^24^ Also, several studies have reported that BAs repress gluconeogenesis through inhibition of phosphoenolpyruvate carboxykinase, glucose‐6‐phosphatase, and fructose 1,6‐bis phosphatase gene expression, through FXR‐dependent and FXR‐independent mechanisms.^22^ However, these findings are somewhat controversial and other pathways may also be involved.^25^ Of note, FXR and its agonist, CDCA, may control the expression of glucose transporter 4 and thereby affect systemic glucose homeostasis.^22^ Furthermore, Zhang et al. observed increased hepatic glycogen synthesis in diabetic *db/db* mice after administration of the FXR agonist, GW4064.^23^ This effect may be mediated, in part, by induction of fibroblast growth factor (FGF) 15 (human ortholog, FGF19), which is an atypical FGF that functions as a hormone with direct metabolic actions in liver and distant organs and has been shown to stimulate glycogen synthesis.^26^ Finally, it should also be noted that FXR deficiency may improve, rather than worsen, glucose homeostasis in some mouse models of obesity.^27^ Thus, BA‐mediated regulation of hepatic glucose metabolism is complex, although most studies support the concept of restitution of insulin sensitivity when FXR is activated. More studies, especially in humans, are warranted.

A role for FXR in regulating lipid metabolism has been also delineated in recent years.^21^, ^22^, ^28^ FXR‐KO mice exhibit a proatherogenic lipoprotein profile with markedly elevated serum and hepatic cholesterol and triglycerides.^20^ Also, administration of an FXR agonist decreased plasma cholesterol, triglyceride, and free fatty acid (FA) levels in *db/db* and wild‐type mice, but not FXR‐KO mice.^23^ Several mechanisms underlie the triglyceride‐lowering effect of FXR agonists, including inhibition of sterol‐regulatory element‐binding protein 1c (SREBP‐1c) expression by a pathway involving the atypical nuclear receptor small heterodimer partner (SHP)^29^ and FXR‐dependent induction of peroxisome proliferator‐activated receptor alpha (PPARα).^22^, ^28^ Thus, activation of FXR represses hepatic *de novo* lipogenesis and stimulates FA β‐oxidation, limiting hepatic lipid accumulation. In addition, FXR regulates several other key genes related to triglyceride metabolism, such as the microsomal triglyceride transfer protein, the very‐low‐density lipoprotein (VLDL) receptor, syndecan‐1 (a protein that binds remnant particles before their transfer to receptors), the FA transporter, CD36, and the apolipoproteins (Apo), CII and CIII.^28^ As a result, FXR can promote plasma VLDL triglyceride clearance by inducing expression of ApoCII, an activator of lipoprotein lipase (LPL), and suppressing expression of ApoCIII, an inhibitor of LPL activity.^21^, ^28^

Activation of FXR in the ileal enterocytes after active intestinal BA uptake also has important metabolic implications through FXR‐stimulated local production of FGF15 (FGF19 in humans).^30^ In hepatocytes, FGF15/19 binds the FGF receptor 4 (FGFR4) and functions as a major regulator of BA synthesis by directly inhibiting cholesterol 7α‐hydroxylase (CYP7A1) expression.^26^ In addition, FGF15/19 also decreases hepatic lipogenesis and indirectly stimulates mitochondrial FA oxidation.^26^, ^28^

FXR also modulates cholesterol homeostasis through several complex inter‐related mechanisms. Hepatic FXR activation negatively regulates expression of CYP7A1, the rate‐limiting enzyme for cholesterol conversion to BAs,^15^ which leads to increased levels of cholesterol within the hepatocyte, down‐regulation of low‐density lipoprotein (LDL) receptor (LDLR), and increased serum LDL cholesterol levels.^22^, ^28^ Additionally, FXR activation may influence reverse cholesterol transport through up‐regulation of scavenger receptor class B type I (SR‐BI), the high‐density lipoprotein (HDL) receptor^31^ and cholesteryl ester transfer protein expression, and by decreasing expression of ApoA1, the major protein component of HDL.^21^ Also, FXR activation may influence HDL remodeling by modulating expression of phospholipid transfer protein and hepatic lipase.^28^ Other data show that BAs may enhance LDLR gene expression through FXR‐dependent, but also FXR‐independent, mechanisms.^32^ Moreover, BAs can decrease hepatic pro‐protein convertase subtilisin/kexin 9 gene expression, a protein that inhibits LDLR activity in a posttranscriptional manner and promotes intracellular degradation of the LDLR.^21^ Thus, BA signaling from intestine and liver significantly impact cholesterol metabolism with ramifications that may limit BA‐based therapies.

## Bile Acids and TGR5: Implications for Energy Metabolism and Inflammation

The discovery of the G‐protein‐coupled bile acid receptor 1 (GPBAR1, also known as Takeda G‐protein‐coupled receptor 5 [TGR5]) in 2002^33^ and the subsequent elucidation of its potential roles in mammalian physiology greatly supported the concept that BAs are signaling molecules with actions in tissues beyond those of the enterohepatic circulation.^34^ TGR5 is widely distributed and expressed by adipocytes, myocytes, immune cells, sinusoidal endothelial cells, bile duct epithelial cells and Kupffer cells, enteroendocrine cells, neurons, and the enteric nervous system with broad physiological and pathophysiological implications.^35^ Relevant for NAFLD/NASH are the roles played by TGR5 in regulating energy expenditure, glucose metabolism, and immunity, which are discussed below.

Mouse studies have shown that activation of TGR5 in brown adipose tissue (BAT) and muscle positively regulates energy expenditure. This occurs through activation of thermogenesis in BAT and muscle by up‐regulation of the gene encoding type 2 iodothyronine‐deiodinase (D2), which converts inactive thyroxine (T4) to active 3,5,3'‐tri‐iodothyronine (T3), resulting in increased oxygen consumption and energy expenditure.^34^, ^35^ Studies in humans have found that serum BA levels correlate with energy expenditure and that short‐term treatment of healthy female subjects with CDCA increased BAT activity and whole‐body energy expenditure.^36^

TGR5 signaling is also involved in glucose homeostasis through its action in enteroendocrine L cells in the intestine.^37^ TGR5 activation in these cells induces release of glucagon‐like peptide‐1 (GLP‐1), an incretin hormone that is secreted by L cells in both ileum and colon in response to luminal nutrients, such as carbohydrates and fats.^38^, ^39^ In the pancreas, GLP‐1 increases insulin synthesis and release, and functions to preserve pancreatic β‐cells by inhibiting β‐cell apoptosis and stimulating β‐cell proliferation.^40^ Moreover, GLP‐1 decreases appetite and food intake through yet‐unclear mechanisms.^39^ Although carbohydrates are believed to be the major stimulus for GLP‐1 release in the intestine, work by Thomas et al.,^37^ using a combination of pharmacological and genetic gain‐ and loss‐of‐function approaches *in vivo*, demonstrated that luminal BAs also stimulate TGR5 signaling, resulting in intestinal GLP‐1 release, and GLP‐1‐associated improvements in glucose tolerance and liver and pancreatic function in obese mice. Interestingly, recent evidence suggests that TGR5 is also expressed by pancreatic β‐cells, where it may regulate insulin secretion.^41^

Given that TGR5 is expressed in mononuclear cells, including Kupffer cells, the resident macrophages of the liver, its modulation has implications for inflammatory conditions. Activation of TGR5 in these cells appears to induce potent anti‐inflammatory effects through inhibition of nuclear translocation of nuclear factor kappa B and suppression of cytokine production.^11^, ^42^ In addition, mouse studies have shown that TGR5 modulates macrophage polarization and infiltration in both adipose tissue and liver, thus diminishing metabolic inflammation.^42^

## BA Transporters and NAFLD

The major carriers responsible for maintaining efficient enterohepatic circulation of BAs have been identified and include members of the ATP‐binding cassette (ABC) and solute carrier (SLC) families of membrane transporters.^13^ The list of carriers include the Na^+^‐taurocholate cotransporting polypeptide (SLC10A1) and bile salt export pump (BSEP; ABCB11) expressed on the sinusoidal and canalicular membranes, respectively, of the hepatocyte, and the apical sodium‐dependent bile acid transporter (ASBT; SLC10A2) and heterodimeric organic solute transporter (OST), OSTα‐OSTβ (OSTα, SLC51A; OSTβ, SLC51B) expressed on the apical brush border and basolateral membranes, respectively, of the ileal enterocyte.^13^, ^14^ These transporters function to conserve and compartmentalize BAs, maintaining high concentrations within the intestinal and hepatobiliary tracts and restricting systemic exposure. Given the above‐mentioned metabolic effects of BA, changes in their enterohepatic cycling and distribution could affect glucose and lipid homeostasis and therefore be relevant for NAFLD. Of note, BA sequestrants, that block intestinal reabsorption of BAs, have been shown to affect cholesterol, triglyceride, and glucose homeostasis in animal models and humans^11^ and have been used therapeutically to treat hypercholesterolemia and T2DM.^43^ However, a comprehensive understanding of the metabolic effects of altered enterohepatic cycling of BAs is lacking. Similarly, studies of the interaction between BA enterohepatic cycling and the pathogenesis of NAFLD are fragmentary and somewhat contradictory. For example, in obese rodents with fatty liver, BA sinusoidal transport seems to be preserved and canalicular BA transport reduced, resulting in mild cholestasis.^44^, ^45^ Consistent with this observation, hepatic overexpression of BSEP prevents hepatic lipid accumulation in mice.^46^ However, other reports have found the contrary.^47^ In humans, studies assessing the expression of BSEP in NAFLD are limited,^48^ and there is no strong human genetic evidence, genome‐wide association studies or candidate gene studies, supporting a role of ABCB11 in NAFLD susceptibility or progression.^49^ Of note, in one study involving 358 NAFLD patients, Iwata et al. searched for an association between advanced liver fibrosis and a common polymorphism in the ABCB11 gene with negative results.^50^ Thus, further studies are needed to clarify the role of hepatic canalicular BA transporters in NAFLD.

With regard to the uptake and efflux transporters of the ileal enterocyte (ASBT, OSTα‐OSTβ), these carriers could play metabolic roles by controlling BA flux and influencing intracellular BA levels and BA signaling in the enterocyte. In light of the recent data pointing to important metabolic effects of FXR activation in the intestine,^51^ the existence of gut‐liver signaling pathways, including the FXR‐FGF15/19 pathway (reviewed in Ferrebee and Dawson^30^), and the recently described intestinal FXR‐ceramide axis,^51^ changes in expression and/or function of ileal BA transporters may be relevant for NAFLD/NASH.

## Influence of Microbiota on BA Metabolism and Its Implications for BA Signaling in NAFLD

The explosion of information regarding the influence of gut microbiota (GM) on human biology is reshaping our understanding of the mechanisms controlling metabolism and energy expenditure with clear implications for obesity, metabolic syndrome, and associated diseases. In the case of NAFLD/NASH, disruption of bacterial gut community (also termed “dysbiosis”) has been linked with disease development and progression although the exact mechanism(s) at play remain unknown.^52^, ^53^, ^54^ BA‐related pathways may be involved given that the GM, through defined enzymatic activities (such as deconjugation, dehydroxylation, oxidation, and epimerization, among others), is a critical modulator of BA pool size and composition and can significantly modify the chemical and signaling properties of BAs.^55^, ^56^ On the other hand, BAs also shape the intestinal microbiome through direct antimicrobial effects, FXR‐induced production of antimicrobial peptides such as angogenin1, by acting as sources of nutrients or reducing potential and by stimulating spore germination.^57^, ^58^ Studies of germ‐free wild‐type and Fxr‐deficient mice have shown that GM‐related changes in BA composition and altered FXR signaling predispose to obesity and obesity‐associated phenotypes, including NASH/NAFLD. Of note, germ‐free mice are resistant to high‐fat diet (HFD)‐induced obesity and GM promotes weight gain and hepatic steatosis through FXR‐dependent mechanisms.^58^ Interestingly, germ‐free mice exhibit a larger BA pool than conventionally raised animals, which has been related to FXR antagonism mediated by tauro‐β‐muricholic acid in the terminal ileum, leading to reduced expression of Fgf15 and higher activity of C7a1 in the liver.^59^ These changes may contribute to resistance of these mice to diet‐induced obesity and suggest that a BA pool size relates to energy expenditure, insulin resistance, and accumulation of triglycerides in the liver under certain feeding conditions, such as an HFD.^60^ However, it must be noted that the relevance of these findings for the human disease remains unclear given that 6‐hydroxylated BAs, such as muricholic acid and its derivatives, are typically not present in humans under normal physiological conditions.^61^ Also, whereas the GM in mice and humans is similar at the genus level, it is unclear how gut bacterial species differences between mouse and human affect microbial BA metabolism. Thus, further research in humans is needed to understand the complex relationship between GM, BAs, and NAFLD/NASH pathogenesis.

## Perspectives on Targeting BA‐Related Pathways for the Treatment of NAFLD

Given the impact of FXR and TGR5 signaling on lipid and glucose metabolism, modulators of these receptors and/or agents that influence endogenous BA levels (i.e., BA transporters modulators or BA sequestrants) could have beneficial therapeutic effects in NAFLD/NASH (Fig. 1). In fact, emerging evidence from mouse and human studies suggests that modulation of FXR (either in the liver or the intestine) and/or TGR5 may be useful in NAFLD treatment by enhancing insulin secretion and sensitivity, inhibiting lipogenesis, and stimulating oxidation of FAs. Moreover, studies showing that circulating BA levels increase along with the metabolic benefits after bariatric surgery,^62^ and that BAs might be involved in NAFLD/NASH reversion in this setting^63^ support the concept that variations in serum BA levels and/or composition have relevant metabolic effects. Of note, changes in circulating BAs with potential benefit for NAFLD/NASH can be achieved through modulation of BA transporters or manipulation of GM.^11^, ^28^ Existing data on targeting BA‐related pathways for NAFLD treatment is summarized below.

## FXR Agonists/Antagonists

FXR agonism has shown benefit in several preclinical models of NAFLD/NASH (see previous works^22^, ^28^ and references therein for details) attributed its metabolic actions as well as its immunomodulatory and anti‐inflammatory effects. This has been demonstrated using natural ligands (i.e., CA or CDCA), semisynthetic modified BAs (i.e., obeticholic acid [OCA]) or synthetic nonsteroidal molecules (i.e., GW4064 and WAY‐362450).^64^ Thus, administration of FXR agonists, such as OCA, GW4064, or CA, are protective against development of liver steatosis and insulin resistance in obese rodents.^22^, ^28^, ^29^ The synthetic FXR agonist, WAY‐362450, also had antisteatotic effects in high‐fructose‐diet‐fed mice, improving intestinal barrier function and reducing hepatic levels of perilipin 2, a lipid droplet protein highly expressed in human and experimental NAFLD.^65^ The same agonist has been shown to attenuate inflammation and fibrosis in experimental NASH.^66^ Collectively, these data suggest that FXR agonism induces metabolic effects that contribute to reduced steatosis and inflammation in experimental NAFLD/NASH. The direct effects on hepatic fibrosis are more controversial given that discrepant data have been published.^22^, ^67^ Of note, it has been reported that the long‐term use of a synthetic FXR agonist can be associated with body weight gain and glucose intolerance as well as worsening of hepatic steatosis.^60^ These effects are possibly related to reduction of the BA pool and energy expenditure and are not observed with natural ligands or BA‐derived FXR agonists.

Human studies with FXR agonists are ongoing, and, at the present time, only two trials have been published, both with the BA‐derivative, OCA. Mudaliar et al. first showed that administration of OCA to patients with NAFLD and T2DM improved insulin resistance and decreased liver fibrosis markers.^68^ More recently, the FLINT (**F**XR **Li**gand Obeticholic Acid in **N**ASH **T**reatment; clinicaltrials.gov identifier NCT01265498,) trial,^69^ which included NASH patients treated with OCA [6α‐ethyl‐CDCA], a potent FXR agonist (25 mg/day for 72 weeks), provided information on the metabolic effects of FXR agonism on glucose and lipid metabolism in humans. In this study, patients treated with OCA showed increased serum levels of insulin and a higher homeostasis model assessment (HOMA) index, a finding that disagrees with previous studies where short‐term administration of OCA improved insulin sensitivity in diabetic patients.^68^ It should be noted that the HOMA index is an imperfect method to assess insulin resistance in diabetic patients,^70^ which ideally should be assessed using a hyperinsulinemic euglycemic clamp. At this time, it is unclear whether the apparently contradictory findings are related to the methods used to assess insulin resistance or whether there are adaptive changes in glycemic control with long‐term use of OCA.

Treatment with OCA significantly improved the primary histological outcome (i.e. improvement of the NAFLD Activity Score [NAS]) and led to a significant reduction of liver fibrosis compared to those of patients treated with placebo. However, NASH resolution occurred in only 22% of patients treated with OCA and no effect was observed in patients with advanced fibrosis. These trends were also observed in a second phase IIa randomized, clinical trial carried out in Japanese patients.^9^ Thus, further studies are needed to better define the clinical usefulness of OCA in NASH given that it remains unproven whether the reported changes in the degree of fibrosis and NAS translate into beneficial effects in terms of survival.^71^

Regarding safety issues, OCA was generally well tolerated. An adverse event occurring more frequently in patients receiving OCA was pruritus, which developed in 23% of patients on OCA and 6% in the placebo group. This symptom rarely led to drug discontinuation. Patients receiving OCA also exhibited increased total serum cholesterol and LDL‐cholesterol levels and a modest, but significant, reduction in HDL‐cholesterol. A reduction in HDL‐cholesterol in the absence of changes in LDL‐cholesterol levels was also noted in primary biliary cholangitis patients treated with OCA plus UDCA.^72^ These plasma lipid changes are likely secondary to increased FGF19 production, which acts to suppress hepatic BA synthesis and hepatic demand for cholesterol.^15^ Of note, the reduction of HDL‐cholesterol could be related to up‐regulation of the HDL‐receptor, SR‐B1, and increased reverse cholesterol transport and as such is not necessarily proatherogenic.^73^, ^74^ The significance of the observed changes in the serum lipid profile on cardiovascular outcomes needs to be explored in more detail as well as the best approach to manage dyslipidemia in any OCA‐based therapeutic strategy.^9^, ^71^

Finally, concerns have been raised regarding the chronically increased levels of FGF‐19 resulting from FXR agonism. This is based in observations showing promitogenic actions of FGF15 (mouse ortholog of FGF19) and FGF19 on HCC development in FGF15 and FGF19 transgenic mice.^75^, ^76^ Also, a role of endogenous Fgf15 in hepatocarcinogenesis in mouse models has been recently demonstrated.^77^ Indeed, the FGF‐19/FGFR4 pathway seems to be of prognostic importance in human liver cancer.^78^ Thus, ileum‐derived FGF15/FGF19 exhibits the potential of being an oncogenic driver and could contribute to HCC development in the setting of chronic liver injury, but more data are needed to understand the metabolic and mitogenic actions of FGF15/FGF19 signaling.

In addition to OCA, other FXR agonists are being investigated in ongoing clinical trials. For example, the synthetic FXR agonist Px‐102/Px‐104^79^ (and the follow‐up compound GS‐9674) is being tested in a phase IIa randomized, clinical trial in patients with NAFLD (ClinicalTrials.gov identifier: NCT01999101), and the selective and highly potent WAY‐362450/FXR‐450 compound is in early‐phase clinical development (ClinicalTrials.gov identifier: NCT00499629). Indeed, this is a very active area, and other agents that will likely emerge as novel FXR modulators continue to be developed.^80^ A recent mouse study using an intestinal‐restricted FXR agonist, fexaramine, also raises questions regarding the optimal target tissue for FXR agonism to treat metabolic disease.^81^ Finally, recent findings from Correia et al.^82^ describing relevant differences among the hepatic FXR isoforms (α1 and α2) with regard to the mechanisms by which they limit hepatocellular lipid accumulation (i.e., with each variant regulating a distinct gene set and Fxrα2 more robustly decreasing hepatic triglyceride levels) could be important to further improve the therapeutic efficacy of FXR agonists.

Although somehow contradictory with the aforementioned data, FXR antagonism could also be of benefit in NAFLD. The intriguing observation that FXR deficiency has beneficial effects on body weight development and glucose homeostasis,^27^ coupled with emerging data related to intestinal FXR antagonism, raises an important question regarding whether the effects of FXR modulation could be tissue specific. In fact, most of the beneficial actions of FXR agonists are likely to depend on their hepatic effects. Conversely, a series of recently published studies provides compelling evidence suggesting that antagonism of intestinal FXR signaling improves metabolic parameters, including NAFLD, in mouse models of obesity.^51^, ^83^ Intestinal FXR antagonism is mediated by muricholic acid derivatives, which are generated by GM and act as naturally occurring FXR antagonist in the ileum.^59^ The underlying mechanisms at play remain unclear, but may be related to the existence of an “intestinal FXR/ceramide axis” whereby ileal production of ceramides controls the hepatic lipogenic program by influencing SRBEP1c.^83^ These studies also underscore the above‐mentioned critical role of GM as modulators of BA pool size and composition and therefore of its action on BA receptors.^9^, ^59^ More studies are needed to determine whether intestinal FXR antagonism can be therapeutically exploited in NAFLD/NASH.

## TGR5 Agonism and TGR5/FXR Dual Agonists

Selective targeting of TGR5 represents an attractive therapeutic approach for NAFLD given that receptor agonism could improve glucose homeostasis and limit body weight gain as well as modulate liver injury in NAFLD/NASH.^11^ Although development of this class of agents initially faced difficulties related to the relatively low binding affinity of BAs for TGR5, the use of medicinal chemistry approaches, as well as screening libraries of plant extracts, has yielded more than a dozen compounds with potent TGR5 activity.^84^ The CA derivative, 6α‐ethyl‐23(S)‐methylcholic acid (INT‐777), was developed as a selective TGR5 agonist and when given to HFD‐fed mice increased energy expenditure and reduced weight gain.^85^ Also, INT‐777 treatment improved liver enzyme levels and ameliorated hepatic steatosis in mice.^37^ However, obtaining specific TGR5/FXR‐specific modulation is challenging given that BA‐ or steroid‐related compounds selective for one receptor may retain a weak affinity for the other,^86^ or *in vivo* metabolism may convert a “specific” agonist for TGR5 or FXR to one with mixed activity toward both. It may also be therapeutically advantageous to develop an agonist with appreciable activity toward both FXR and TGR5. McMahan et al. found that treating *db/db* obese mice with one such dual FXR/TGR5 agonist, INT‐767, decreased hepatic steatosis, reduced proinflammatory cytokine expression, and shifted the monocyte and macrophages toward an anti‐inflammatory M2 phenotype.^87^

Additionally, given that the TGR5 signaling pathway is critical in regulating intestinal GLP‐1 secretion,^37^ its targeting may be beneficial in organ‐specific insulin sensitivity, hepatic lipid handling, and adipose tissue dysfunction. Of note, a recent small, but well‐designed, study showed that administration of the GLP‐1 analog, liraglutide, led to histological resolution of NASH in a significant proportion of subjects.^88^ Moreover, the drug also reduced insulin resistance and decreased hepatic *de novo* lipogenesis and lipotoxicity in key metabolic organs.^89^ Thus, the ability of BA‐based therapies to augment GLP signaling warrants further investigation as part of understanding their potential as a disease‐modifying intervention in NASH.

In spite of the promising aforementioned results, the only published human study in which a TGR5 agonist was administered to diabetic patients yielded somewhat disappointing results given that glucose levels were strikingly increased and not reduced as expected.^90^ Further research is needed to determine whether TGR5‐based therapies would be a viable approach to metabolic disease and NAFLD.

## Inhibitors of BA Absorption

As alluded to above, blocking intestinal BA absorption through intestinal BA sequestration improves metabolic aspects of NAFLD in some human and mouse studies, perhaps through TGR5‐related effects.^11^ However, the only study specifically designed to assess the efficacy of colesevelam, a potent BA sequestrant, to decrease liver fat in patients with biopsy‐proven NASH yielded negative results.^91^ On the other hand, in line with the concept that inhibition of intestinal FXR signaling has benefit in NAFLD,^51^ preliminary observations show that pharmacological inhibition of ASBT substantially improves hepatic lipid accumulation in mice fed an HFD and cholesterol.^92^ The therapeutic potential for ASBT blockade to treat NAFLD in patients remains unexplored; however, several ASBT inhibitors are already in development to treat other conditions, such as dyslipidemia, cholestasis, or constipation.^30^

## Ursodeoxycholic Acid and Derivatives

Before the identification of FXR and TGR5 BA receptors, other BAs have been studied as potential treatments for NAFLD/NASH. Ursodeoxycholic acid (UDCA), a BA with immunomodulatory, antioxidant, and antiapoptotic properties is used to treat other forms of liver disease and was considered a reasonable potential therapy for NASH.^8^ Although a considerable number of rodent and human studies have been conducted, the findings with regard to benefit have been conflicting.^28^ Thus, whereas a small trial showed a significant improvement in alkaline phosphatase, alanine aminotransferase, gamma‐glutamyl transpeptidase, and in liver steatosis in NASH patients treated with UDCA for 12 months,^93^ larger studies showed no benefits of UDCA in NAFLD.^94^, ^95^ Finally, a recent study in morbidly obese patients who were randomized to receive UDCA (20 mg/kg/day) or no treatment 3 weeks before bariatric surgery found that UDCA may even function in an FXR antagonistic manner *in vivo*, reducing levels of circulating FGF19, and promoting stearoyl CoA desaturase 1 (SCD1) and lipogenesis in liver and visceral white adipose tissue.^96^ Thus, UDCA efficacy for NAFLD/NASH treatment remains unproven. Of note, a side‐chain shortened derivative of UDCA, norUDCA, with distinct metabolic and signaling properties has been shown to improve NASH in mouse models,^97^ and a multicenter, double‐blind, randomized, placebo‐controlled, phase II dose‐finding trial has been initiated to compare different doses of norUDCA with placebo in the treatment of NAFLD (EudraCT Number: 2013‐004605‐38).

## FA/BA Conjugates

Aramchol is a novel synthetic lipid molecule obtained by conjugating two natural components, CA and arachidic acid (a saturated FA), through a stable amide bond. This FA/BA conjugate inhibits SCD1 activity and activates cholesterol efflux by stimulating the ABC transporter, A1, a pan cellular cholesterol export pump.^98^ In a phase 2 study involving 60 patients with biopsy‐proven NAFLD, treatment with 100 or 300 mg/day of aramchol for 3 months significantly reduced liver fat content in a dose‐dependent manner and was associated with a trend of metabolic improvements.^99^ A large‐scale, multicenter, phase IIb, randomized, double‐blind, placebo‐controlled study designed to evaluate the efficacy and safety of Aramchol in NASH is ongoing (ClinicalTrials.gov Identifier: NCT02279524).

## Modulation of Gut Microbiota

As mentioned above, the GM is responsible for a myriad of chemical modifications of BAs that can modify both BA pool size and BA signaling properties.^56^ Thus, therapeutic interventions targeting GM in NAFLD/NASH may, at least partially, act through induction of changes in host BA profiles. Nutraceutical (diet, probiotics), pharmaceutical (antibiotics, tempol, and fexaramine), or surgical (bariatric surgery) interventions may modify obesity and obesity‐associated metabolic phenotypes, including NAFLD/NASH, through changes in BA composition that influence signaling through FXR and likely other BA receptors.^62^, ^81^, ^83^, ^100^

## Conclusions

Given that recent studies in humans and mice indicate that BA signaling may play central roles in the development, progression, and regression of the metabolic abnormalities at play in NAFLD/NASH, specific targeting of BA‐related pathways at the level of hepatocyte, intestine, colon, BAT, or other sites holds promise for disease management. Although preclinical and available clinical studies are promising, developing safe and effective BA‐based therapies still faces many challenges, including FXR/TGR5 selectivity and tissue‐specific activity of compounds as well safety issues, particularly those related to the long‐term use of a given agent.

Author names in bold designate shared co‐first authorship.
